# Draft Genome Sequence of *Marinobacter* sp. Strain ANT_B65, Isolated from Antarctic Marine Sponge

**DOI:** 10.1128/genomeA.01404-17

**Published:** 2018-01-04

**Authors:** Paula de França, Esther Camilo, Fabiana Fantinatti-Garboginni

**Affiliations:** a Multidisciplinary Center for Chemical, Biological and Agricultural Research, University of Campinas, Paulínia, São Paulo, Brazil; b Graduate Program in Genetics and Molecular Biology, Institute of Biology, University of Campinas, Campinas, São Paulo, Brazil; c Motiva Bioinformática ME, Sertãozinho, São Paulo, Brazil

## Abstract

*Marinobacter* sp. strain ANT_B65 was isolated from sponge collected in King George Island, Antarctica. The draft genome of 4,173,840 bp encodes 3,743 protein-coding open reading frames. The genome will provide insights into the strain’s potential use in the production of natural products.

## GENOME ANNOUNCEMENT

The genus *Marinobacter* comprises 41 species of Gram-negative bacteria isolated from marine and saline environments. *Marinobacter* species can survive in different conditions, such as oil-polluted saline soil (1) and hydrothermal sediment (2), and they can be psychrotolerant (3) or psychrophilic (4).

*Marinobacter* sp. strain ANT-B65 was isolated from sponge collected in Admiralty Bay, King George Island, Antarctica (62°05,130′S 58°23,356′W), during an expedition in the austral summer of 2010. Strain ANT_B65 was isolated using M1 medium (10 g · liter^−1^ soluble starch, 4 g · liter^−1^ yeast extract, 2 g · liter^−1^ peptone, and 15 g · liter^−1^ agar) prepared with artificial sea water (0.1 g · liter^−1^ KBr, 23.48 g · liter^−1^ NaCl, 10.61 g · liter^−1 ^MgCl_2_-6H_2_O, 1.47 g · liter^−1 ^CaCl_2_-2H_2_O, 0.66 g · liter^−1^ KCl, 0.04 g · liter^−1 ^SrCl_2_-6H_2_O, 3.92 g · liter^−1 ^Na_2_SO_4_, 0.19 g · liter^−1 ^NaHCO_3_, and 0.03 g · liter^−1 ^H_3_BO_3_) at the Multidisciplinary Center for Chemical, Biological and Agricultural Research, University of Campinas (Campinas, São Paulo, Brazil).

A paired-end library was sequenced using the HiSeq 2500 system and yielded 8,229,346 reads of 251 bp. The reads were assembled with SeqMan NGen version 14.0 software (DNASTAR, Inc., WI, USA), resulting in a total genome length of 4,173,840 bp in 8 contigs, with an *N*_50_ of 2,216,721 bp and a GC content of 53.7%.

The genome was annotated using Prokka (5), the NCBI Prokaryotic Genome Annotation Pipeline (PGAP), and the Rapid Annotations using Subsystems Technology (RAST) server version 4.0 (6, 7). Prokka identified 3,817 coding sequences (CDSs), 25 tRNAs, 5 rRNAs, and 25 miscellaneous RNAs. PGAP identified 3,743 protein-coding genes, 44 tRNAs, 12 rRNAs, and 580 pseudogenes. RAST identified 3,959 CDSs, 45 tRNAs, 12 rRNAs, and 443 subsystems and predicted that 3 proteins are phage components.

RAST and the Kyoto Encyclopedia of Genes and Genomes (KEGG) identified secondary metabolites related to an auxin biosystem. The antiSMASH v.4.0 (8) analysis revealed one known secondary metabolite cluster, an ectoine. This is a natural compound used by the microorganism to survive in osmotic stress and found mainly in halophilic bacteria (9, 10). The NaPDos (11) analysis revealed four gene clusters related to the ketosynthase domain, three gene clusters related to fatty acid synthesis, and one gene cluster related to a spinosad. This cluster was associated with a macrolide produced by *Saccharopolyspora spinosa* (12) and used in agriculture as a potent insecticide.

The genome of *Marinobacter* sp. ANT_B65 provides insights into its potential use in the production of natural products.

### Accession number(s).

This whole-genome shotgun project has been deposited at DDBJ/ENA/GenBank under the accession number NXGV00000000. The version described in this paper is the first version, NXGV01000000.
